# How Victim Sensitivity leads to Uncooperative Behavior via Expectancies of Injustice

**DOI:** 10.3389/fpsyg.2015.02059

**Published:** 2016-01-13

**Authors:** Simona Maltese, Anna Baumert, Manfred J. Schmitt, Colin MacLeod

**Affiliations:** ^1^Department of Psychology, University of Koblenz-LandauLandau, Germany; ^2^School of Psychology, University of Western AustraliaPerth, WA, Australia

**Keywords:** expectancy tendencies, cooperation, trust, justice sensitivity, information processing

## Abstract

According to the Sensitivity-to-mean-intentions model, dispositional victim sensitivity involves a suspicious mindset that is activated by situational cues and guides subsequent information processing and behavior like a schema. Study 1 tested whether victim-sensitive persons are more prone to form expectancies of injustice in ambiguous situations and whether these expectancies mediate the relationship between victim sensitivity and cooperation behavior in a trust game. Results show an indirect effect of victim sensitivity on cooperation after unfair treatment (vs. control condition), mediated by expectancies of injustice. In Study 2 we directly manipulated the tendency to form expectancies of injustice in ambiguous situations to test for causality. Results confirmed that the readiness to expect unjust outcomes led to lower cooperation, compared to a control condition. These findings provide direct evidence that expectancy tendencies are implicated in elevated victim sensitivity and are of theoretical and practical relevance.

## Introduction

People differ systematically in their perceptions of as well as their emotional and behavioral reactions to injustice (Schmitt, 1996). Social justice research has provided intriguing evidence that individual differences in dispositional justice sensitivity (JS) are associated with reactions to injustice (Schmitt et al., 2005, 2010). For example, JS has been found to shape anger, protest, and retaliation in reaction to own disadvantages (Schmitt and Dörfel, 1999). One important dimension of JS is the intolerance of unfair treatment directed toward the self, namely victim sensitivity. Strikingly, high victim sensitivity was found to be associated with reduced willingness to cooperate (Gollwitzer et al., 2009). For example, highly victim-sensitive persons were found to refrain from displaying solidarity with disadvantaged others (Gollwitzer et al., 2005).

The Sensitivity-to-mean-intentions (SeMI) Model was proposed to explain this and similar antisocial effects of victim sensitivity by delineating the underlying processes that translate this disposition into uncooperative behavior (Gollwitzer and Rothmund, 2009; Gollwitzer et al., 2013). This account assumes that victim-sensitive persons have a generalized expectancy that others harbor mean intentions. More precisely, the model proposes that victim-sensitive persons are characterized by a suspicious mindset that is activated by contextual cues suggesting the intentional meanness of others. Assumedly, the activated mindset guides information processing and behavior like a schema and consists of three components: hostile interpretations, the motivation to avoid exploitation, and cognitions legitimizing one’s own antisocial behavior. Here we focus on hostile interpretations, and more specifically, on expectancies of injustice in ambiguous situations.

Until now, there has been only indirect evidence that victim-sensitive persons have heighted expectancies of others mean intentions, and that this processing pattern is responsible for their reluctance to cooperate in socially uncertain situations. We argue that it is crucial to assess such expectancies in order to test whether they are indeed characteristic of persons high (compared to low) in victim sensitivity, and directly manipulate these expectancies in order to determine whether they causally contribute to reduced behavioral cooperation when the suspicious mindset is activated.

This research will enable a more detailed understanding of victim sensitivity by clarifying the processes responsible for its detrimental social effects. Adopting a social-cognitive approach makes it possible to move beyond the description of interindividual differences toward explanation of the mechanisms that give rise to phenomena, such as the withdrawal of cooperation (Baumert and Schmitt, 2012). The resulting knowledge can inform the development of effective interventions capable to enhance adaptive behavior in high victim-sensitive persons, and so offers the promise of yielding applied benefits.

### Expectancy of Injustice

The availability of hostile interpretations is seen as core to the suspicious mindset in victim-sensitive persons. As described by Fein and Hilton (1994): “after the activation of a suspicious mindset, even slight or meaningless incidents become likely to be interpreted as evidence for this person’s mean intentions” (cited by Gollwitzer et al., 2013, p. 418). Moreover, in situations where outcomes are uncertain, the activated suspicious mindset triggers the expectancy that one will be unjustly disadvantaged (Gollwitzer et al., 2013).

Indirect evidence that expectancies of injustice characterize heightened victim sensitivity comes from studies assessing behavior in uncertain social situations. An often-used paradigm that includes such a situation is the so-called trust game (Berg et al., 1995). In an adapted version of this game, participants are faced with two financial decisions in the role of anonymous Persons A and B. Persons A and B receive an equal amount of money from the experimenter. First, Person A is free to invest any amount by transferring it to Person B. This investment is then tripled by the experimenter. Second, Person B has two options: to keep the tripled investment of Person A or to transfer back a share of this investment to Person A such that both persons will have the same outcome (Gollwitzer and Rothmund, 2011).The first decision in the role of Person A is a situation in which the participant’s cooperation can be exploited by an anonymous partner in the role of Person B. Thus, non-cooperation of the Person A is taken to indicate the expectancy that Person B will be uncooperative. The second decision in the role of Person B is a situation, where participants are able to exploit the cooperativeness of their anonymous partner in order to gain as much money as possible. The focus in the present research is on the decision in the role of Person A which will be termed cooperation decision in the remainder of this paper.

Gollwitzer and Rothmund (2011, Study 2) used this version of the trust game and found that victim-sensitive persons reduced their cooperation in the role of Person A, when they had previously been confronted with the selfish behavior of a different interaction partner in an unrelated situation. The authors conclude that the unfair disadvantage suffered prior to the trust game led victim-sensitive persons to expect mean intentions of the new interaction partner, and consequently resulted in lower cooperation rates. These findings are consistent with the idea that victim-sensitive persons have a heightened tendency in ambiguous situations to expect that they will be unfairly treated, in cases when their suspicious mindset was activated by an unrelated situation. However, evidence from this study (as well as similar studies by Gollwitzer et al., 2009 and Rothmund et al., 2011) is limited by the fact that the association between expectancy tendencies and victim sensitivity was not directly assessed, and so no conclusion about the exact functioning of these expectancy tendencies can be drawn.

Baumert et al. (2012) directly assessed expectancy tendencies and found that high (vs. low) victim-sensitive persons exhibited heightened anticipation of unjust (but also just) outcomes in ambiguous situations. Expectancy tendencies were assessed by means of a fragment completion task. High (vs. low) victim-sensitive persons were faster to complete fragments that resolved ambiguous passages to indicate an unjust or just outcome, rather than providing a completion unrelated to justice. In contrast to the assumptions of the SeMI model, the findings of the Baumert et al. (2012) study suggest that victim-sensitive persons readily form expectancies of both unjust and just outcomes in ambiguous situations. However, in this study, a suspicious mindset was not activated by confronting participants with cues of others’ mean intentions prior to the assessment of expectancies. Baumert et al. (2012) speculated that the situational activation of a suspicious mindset may be necessary before the information processing of victim-sensitive persons becomes biased toward expectancies of unjust outcomes.

Thus, there is no direct evidence that expectancy tendencies mediate the relationship between victim sensitivity and reluctance to cooperate in situations when exposure to cues signaling mean intentions permit activation of a suspicious mindset.

### The Present Research

In two studies we investigated the association between victim sensitivity, expectancies of injustice, and reduced cooperation. In Study 1, we adopted a moderated mediation approach to compare effects of victim sensitivity under conditions that did or did not cue activation of a suspicious mindset. We employed a precue procedure that exposed participants to unfair behavior of an ostensible interaction partner (unfairness precue) or to a fair outcome (control condition). Subsequently, we assessed the tendency to form expectancies of unjust and just outcomes and observed cooperative behavior in a trust game. Based on the assumptions outlined above, the experienced unfairness should activate a suspicious mindset in victim-sensitive but not in insensitive persons, and subsequently guide the formation of expectancies of injustice in ambiguous situations, resulting in withdrawal of cooperation in the trust game.

To assess expectancies of injustice, we adapted a recognition paradigm from research on anxiety (Mathews and Mackintosh, 2000). Participants read scenarios that were left ambiguous with regard to the outcome for the narrator. Each scenario was presented with an identifying title. Afterward, participants saw this title again, together with sentences that described unjust and just alternative outcomes for the respective scenario. Participants were required to indicate how well each sentence fit with the previously read scenario. It was assumed that people would tend to resolve the uncertainty while reading a scenario by anticipating a likely outcome (e.g., Simpson, 1981). Consequently, persons with a heightened tendency to expect injustice would anticipate unjust outcomes while encoding the original scenarios. Forming an unjust (just) interpretation of the ambiguous scenario should lead to the endorsement of sentences that described an unjust (just) outcome for the scenario.

We predicted that in the unfair precue condition, but not in the control condition, high (compared to low) victim-sensitive persons would tend to expect unjust outcomes. We anticipated no effect of victim sensitivity on the expectancy of just outcomes. Furthermore, we predicted that under activation of the suspicious mindset the tendency to form expectancies of injustice would mediate the expected relationship between victim sensitivity and behavioral cooperation in the trust game.

Based on the results of Study 1, we experimentally manipulated the tendency to form expectancies of injustice by means of a training procedure in Study 2. This allowed us to test the hypothesized causal role of unjust expectancies in the proposed mediating process for cooperation behavior. In a training procedure that we adapted from research on anxiety (Mathews and Mackintosh, 2000) participants read sentences that described situations permitting potentially unjust or just outcomes. The last words of each sentence resolved this ambiguity by communicating whether an unjust or just outcome occurred. These final words were presented as fragments that the participant had to complete as quickly as possible. In the unjust expectancy training condition, all fragments resolved the ambiguity to indicate an unjust outcome. By contrast, in the control condition, the sentences described situations unrelated to justice and the fragments yielded final words unrelated to justice. After the training procedure, all participants received additional fragments (*probes*) that resolved ambiguous sentences in describing an unjust, just or neutral outcome. The reaction times to complete these fragments served as a measure to estimate the success of the training manipulation.

It was intended that participants in the unjust expectancy training condition would resolve the ambiguity while reading the sentences and anticipate unjust outcomes. This acquired readiness to form expectancies of injustice would lead them to faster reactions (compared to control condition) to solve the probes that yielded an unjust outcome (but not just and neutral outcomes). Furthermore, regarding cooperation in the trust game, we predicted that persons in the unjust expectancy training condition (compared to the control condition) would allocate less money to their partners in the trust game, when in the role of Person A.

### Ethics Approval

These studies were carried out in accordance with the ethics guidelines of the DGPs and the BDP (German Association of Psychology) and approved by the local ethics commission of the University of Landau. Participants provided their informed consent through agreeing in an interactive dialog field.

## Study 1

### Method

#### Sample

Fifty-four undergraduate students (83% female; ages: 19–47 years; *M* = 23.15; *SD* = 5.69) participated in a study ostensibly on verbal abilities. In return for their participation, students received 8€.

#### Procedure

In the first weeks of the semester, students were invited to complete a questionnaire containing personality measures, including the JS scales. Several weeks later, the students were invited to participate in an independent computer-based laboratory experiment. Upon arrival, participants were seated at one of four separated workplaces and randomly assigned to either the unfair precue condition (*n* = 30), or to the control condition (*n* = 24). After providing demographic information, participants had to resolve an anagram task that served as precue procedure and will be explained below. Subsequently, expectancy tendencies were measured with a recognition paradigm and participants made financial decisions in a trust game. Finally, manipulation checks were assessed and participants were fully debriefed, thanked, and dismissed.

#### Materials

All materials were provided in German language. For comprehension we present own English translations.

##### Justice Sensitivity

The Justice Sensitivity Inventory (Schmitt et al., 2010) served to measure victim sensitivity with 10 items (α = 0.90; e.g., “I ruminate for a long time when other people are treated better than me”). The response scales ranged from 0 (*totally disagree*) to 5 (*totally agree*)^1^.

##### Precue procedure

Based on the procedure of Gollwitzer and Rothmund (2011, Study 2), participants worked on a computer-based anagram task for 1 min with an anonymous partner, supposedly participating in the study simultaneously in another room. Participants were instructed that they would receive credits, depending on their and the other participant’s performance, and that the credits would be transferred into raﬄes for the lottery of an iPod among all participants after completion of data collection. Thus, their chances to win the iPod would be higher the more credits they earned. In order to raise the credibility of this procedure, we had two experimenters, one of them changed between the rooms to retrieve the information about the performance of the fictitious partner. However, this information was prepared so that the partners’ performance was similar to that of the participant.

In the unfair precue condition, after learning about their own and their partner’s performance, the participant was asked to decide on the allocation of raﬄes among them. They were told that their partner would take the same decision simultaneously and that both decisions would be averaged to determine the number of raﬄes that each person would receive. One experimenter left the room to retrieve the partner’s decision. Independent of their own decision, participants in the unfair precue condition received the feedback that their fictitious partner had allocated 75% of the raﬄe tickets to him/herself.

By contrast, in the control condition, the whole amount of obtained raﬄes was split equally between the two partners. To visualize the allocation of raﬄes, every participant received a predesigned sheet where the experimenter noted performances and decisions.

##### Assessment of expectancies of injustice

The recognition paradigm to assess expectancies of injustice was presented as a fragment completion task. It consisted of two parts, an encoding phase and a recognition phase. In the encoding phase, participants were instructed to actively take the perspective of the narrator while reading passages with fragmented last words that had to be completed as fast as possible.

They started with 30 fragment completion trials^2^ to get used to the task. Participants were instructed to press a marked button as soon as they knew the correct solution. With the key stroke, they proceeded to the next screen where they typed in the missing letters of the fragmented words. These initial 30 trials were followed by 13 scenarios. The only noticeable difference for the participants was that each of these scenarios was introduced by an identifying title (in contrast to the previous trials). Half of these scenarios^3^ described a situation, where the actual outcome was left uncertain, and the last words of each scenario were fragmented. For example:

“The presentation.

During the last weeks I worked overtime for my boss. Even today, my colleagues went already home, while I still have to to prepare a presentation. Now my boss is calling me for the *ann_al perso_nel ta_k*. (correct fragment completion: annual personnel talk).”

In the recognition phase, for each of the previously read scenarios, the identifying title was displayed together with four descriptions related to the content of the respective scenario. For the relevant scenarios, four alternative outcomes were presented that had been pretested in an independent sample^4^. Two sentences described unjust, and the other two just outcomes for the narrator. One of each type of sentence, the *target*, described an unjust or just disadvantage and contained information that corresponded to the initial scenario. For the above described scenario they read as follows:

Although I am strongly committed, my boss accuses me of insufficient performance. (*unjust target*)

My boss promises me to pay my overtime work in the next month because of my strong commitment. (*just target*)

The other of each type of sentence, the *foil*, described an unjust or just disadvantage as well but contained information that was false in the sense that it contradicted the information given in the initial scenario. For the above described scenario, the foils were:

This week my boss gives me even more work to do and does not invite me to the annual personnel talk. (*unjust foil*)

My colleagues are helping me to prepare the presentation that I am free to go for the annual personnel talk. (*just foil*)

The foils served to disentangle the endorsement of unjust targets, indicating expectancies of injustice, from the endorsement of both, unjust targets and foils, indicating a response bias to unjust outcomes in general. The four outcomes for each scenario were presented one after another in random order that was fixed across participants. Participants were asked to indicate for each sentence separately, how well it matched the content of the corresponding scenario. Response options ranged from 1 (*does not match the scenario at all*) to 6 (*matches the scenario very well).*

##### Trust game

We used the modified trust game employed by Gollwitzer and Rothmund (2011) to assess behavioral cooperation. Participants were told that they would interact with another anonymous, randomly chosen person that participated in the study but was not present in the same session. The task was introduced in the following way on the computer screen: Persons A and B receive 100 ct each and have to take two decisions. First, Person A is free to invest any amount to Person B. This investment is tripled by the experimenter. Second, Person B has two options: to keep the tripled investment of Person A or to share with Person A such that both receive the same outcome. Every participant took both decisions (without knowing about the partner’s decisions), but the dependent variable of interest was the decision in the role of Person A as index of behavioral cooperation. Participants were informed that their decisions were about real money and that, after the end of the study, three participants were chosen randomly to receive their obtained money.

##### Manipulation checks

At the end, participants were asked about their perception of the allocation of the lottery tickets in the anagram task, in order to validate the effectiveness of the precue manipulation of perceived unfair vs. fair treatment. They rated eight items on a scale from 1 (*totally disagree*) to 6 (*totally agree*) to assess fairness (α = 0.76, e.g., “The number of raﬄes I obtained was fair”).

To reiterate our hypotheses in Study 1, we predicted that in the unfair precue condition, but not in the control condition, high (compared to low) victim-sensitive persons would more strongly endorse targets but not foils describing unjust outcomes (Hypothesis 1A). We expected no effect of victim sensitivity on the endorsement of just targets or foils (Hypothesis 1B). Furthermore, we predicted that, in the unfair precue condition (but not in the control condition), there would be an indirect negative effect of victim sensitivity on the amount that each participant transferred in the role of Person A in the trust game, mediated by the endorsement of targets, indicating unjust outcomes (Hypothesis 2).

### Results

Correlations, means, and standard deviations of all variables are reported in **Table 1**, separately for precue conditions. Regarding the cooperation decision in the role of Person A in the trust game, on average, participants transferred 81 ct (*SD* = 22.8) to their interaction partners, with no significant differences between average transfer rates in the unfair precue condition (*M* = 80.0, *SD* = 23.0) and in the control condition (*M* = 82.0, *SD* = 23.0), *t*(52) = -0.35, *p* = 0.73, *d* = 0.09.

**Table 1:** Correlation, mean, and standard deviation of all variables in the unfair precue condition and in the control condition in Study 1.

	(1)	(2)	(3)	(4)	(5)	(6)
**Unfair precue condition**						
(1) Victim sensitivity	1					
(2) Cooperation	-0.31	1				
(3) Unjust target	0.41^∗^	-0.39^∗^	1			
(4) Unjust foil	0.32^†^	-0.16	0.58^∗∗^	1		
(5) Just target	0.07	-0.17	-0.19	-0.36^†^	1	
(6) Just foil	0.05	-0.01	0.13	0.34^†^	0.01	1
*M*	2.70	80.0	2.79	3.55	1.69	1.79
*SD*	0.89	23.3	1.11	1.19	0.89	0.63
**Control condition**						
(1) Victim sensitivity	1					
(2) Cooperation	0.55^∗∗^	1				
(3) Unjust target	-0.21	-0.33^∗∗^	1			
(4) Unjust foil	-0.14	-0.14	0.56^∗∗^	1		
(5) Just target	0.33	0.36^†^	-0.44^∗^	-0.09	1	
(6) Just foil	-0.09	0.27	0.06	0.48^∗^	0.31	1
*M*	2.77	82.2	2.93	3.14	1.52	1.50
*SD*	0.83	22.7	1.10	1.27	0.63	0.69

*For Victim Sensitivity, response scales ranged from 0 (*fully disagree*) to 5 (*fully agree*)*.

*Unjust target; just target = endorsement of unjust/just expectancies. Unjust foil; just foil = response bias. Response scales from 1 (*does not match the scenario at all*) to 6 (*matches the scenario very well*). Cooperation = amount of money (in Cent) transferred by Person A in the trust game*.

*^†^*p* < 0.10, ^∗^*p* < 0.05, ^∗∗^*p* < 0.01*.

#### Manipulation Checks

As expected, participants in the unfair precue condition perceived the allocation of the lottery tickets in the anagram task as significantly less fair (*M* = 3.13; *SD* = 0.97) than participants in the control condition (*M* = 4.51, *SD* = 0.66), *t*(52) = -5.90, *p* < 0.01, *d* = 1.66.

#### Relationship between Victim Sensitivity and Expectancy Tendencies

To test Hypothesis 1, we conducted separate moderated regression analyses, with the endorsement of unjust and just targets and foils as dependent variables, respectively. Condition (dummy-coded: 0 = control condition; 1 = unfair precue condition), z-standardized victim sensitivity, as well as the interaction victim sensitivity × condition were entered as predictors. The dependent variables remained in their metric. Thus, we report semi-standardized *B*-weights.

**FIGURE 1:**
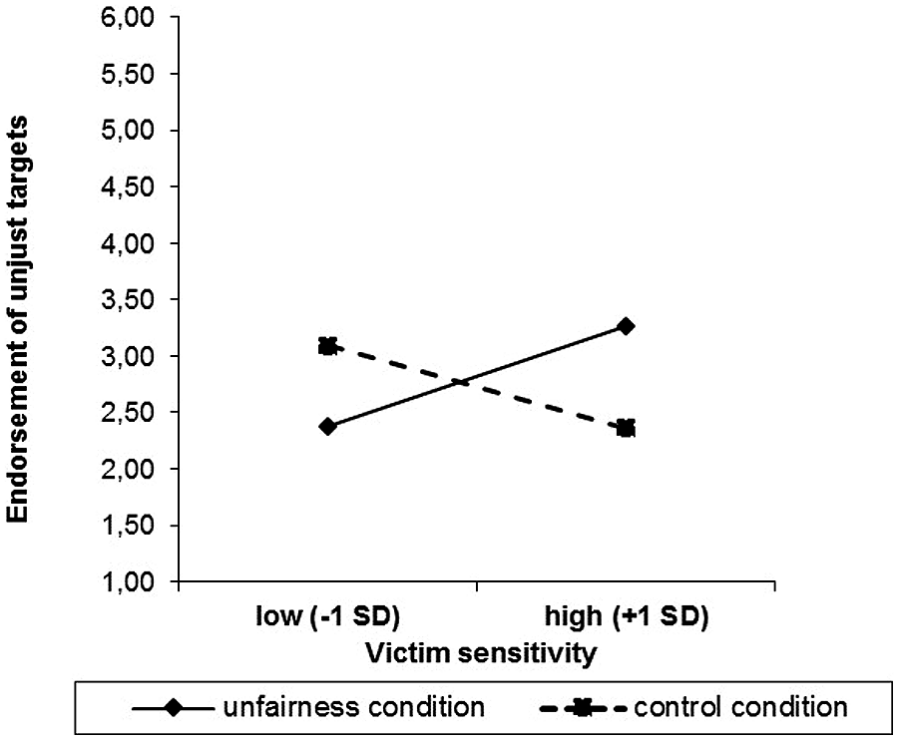
**Endorsement of unjust targets dependent on victim sensitivity and condition**.

Regarding the endorsement of unjust targets, the complete regression model explained 12% of the variance, *F*(3,48) = 2.17, *p* = 0.10. There were no significant main effects of condition, *B* = -0.01, *t*(51) = -0.04, *p* = 0.97, or of victim sensitivity, *B* = -0.27, *t*(51) = -1.00, *p* = 0.32. There was a significant interaction effect of victim sensitivity × condition, *B* = 0.79, *t*(51) = 2.26, *p* = 0.03, Δ*R^2^* = 0.09. In the unfair precue condition, persons high in victim sensitivity endorsed unjust targets significantly more than did persons low in victim sensitivity, *B* = 0.52, *t*(27) = 2.34, *p* = 0.03 (**Figure 1**). In the control condition, there was no significant relationship between victim sensitivity and the endorsement of unjust targets, *B* = -0.27, *t*(22) = -1.00, *p* = 0.33. An *a posteriori* power analysis with *Gpower* (Faul et al., 2009) for the interaction effect revealed *1-*β = 0.62 to detect an increase in *R^2^* of 0.09.

For unjust foils, the complete regression model explained 9% of the variance, *F*(3,48) = 1.60, *p* = 0.20. There were no significant main effects of condition, *B* = 0.20, *t*(51) = 0.92, *p* = 0.36, or victim sensitivity, *B* = -0.11, *t*(51) = -0.52, *p* = 0.60 and no significant interaction effect of victim sensitivity × condition, *B* = 0.43, *t*(51) = 1.65, *p* = 0.11, Δ*R^2^* = 0.05. Although the interaction effect was not significant, it has to be noted that in the unfair precue condition, there was a marginally significant simple slope for the regression of the endorsement of unjust foils on victim sensitivity, *B* = 0.32, *t*(27) = 1.76, *p* = 0.09.

Regarding the endorsement of just targets, the complete model explained 6% of the variance, *F*(3,48) = 0.94, *p* = 0.43. There were no significant main effects of condition, *B* = 0.26, *t*(51) = 0.77, *p* = 0.44, or victim sensitivity, *B* = 0.44, *t*(51) = 1.45, *p* = 0.15 and no significant interaction effect of victim sensitivity × condition, *B* = -0.23, *t*(51) = -0.89, *p* = 0.38, Δ*R^2^* = 0.02. For the endorsement of just foils, the complete regression model explained 4% of the variance, *F*(3,48) = 0.69, *p* = 0.56. There were no significant main effects of condition, *B* = 0.26, *t*(51) = 1.37, *p* = 0.18, or victim sensitivity, *B* = -0.07, *t*(51) = -0.42, *p* = 0.67 and no significant interaction effect of victim sensitivity × condition, *B* = 0.07, *t*(51) = 0.47, *p* = 0.64, Δ*R^2^* = 0.004.

#### Effects of Victim Sensitivity on Cooperation Behavior in the Trust-Game Mediated by Expectancy Tendencies

**FIGURE 2:**
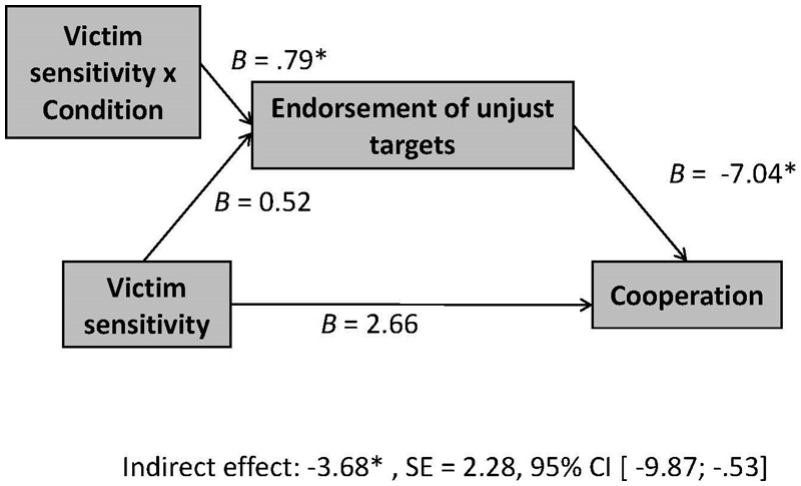
**Moderated mediation model for the interaction victim sensitivity × condition on cooperation in the trust game, showing *B*-weights and indirect effects for endorsement of unjust targets (unjust expectancies) as mediator**. ^∗^*p* < 0.05.

**FIGURE 3:**
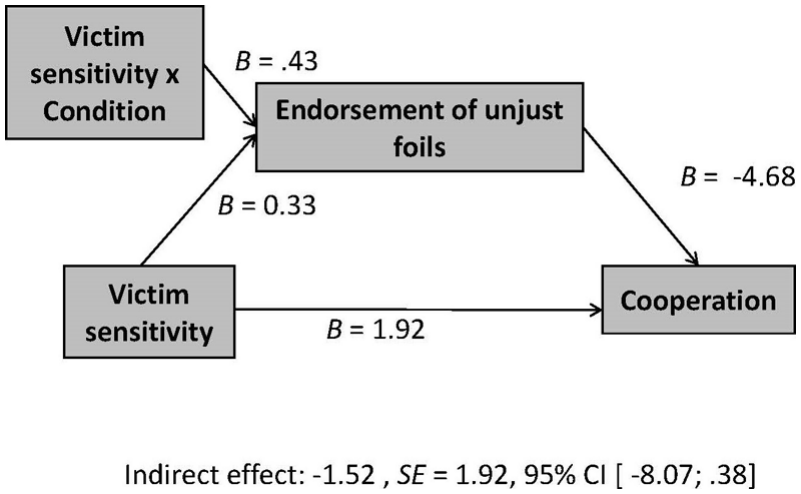
**Moderated mediation model for the interaction victim sensitivity × condition on cooperation in the trust game, showing *B*-weights and indirect effects for endorsement of unjust foils (response bias) as mediator**.

As can be seen in **Table 1**, in the unfair precue condition, there was no significant bivariate correlation between victim sensitivity and behavioral cooperation in the trust game (*r* = -0.31, n.s.). Nevertheless, using the process tool for SPSS (Hayes, 2013), we conducted moderated mediation analyses in order to test for an indirect effect of victim sensitivity on cooperation behavior via expectancy tendencies, which we expected in the unfair precue condition (Hypothesis 2). Further, we wanted to distinguish the formation of expectancies of injustice from a response bias regarding these processes’ potential relevance for behavioral reactions. Therefore, we tested two moderated mediation models. In both models victim sensitivity (z-standardized) served as independent variable, condition (dummy-coded: 0 = control condition; 1 = unfair precue condition) as moderator, and investment in the role of Person A in the trust game as the dependent measure of behavioral cooperation. In the first model, the endorsement of unjust targets was tested as mediator, in the second model the endorsement of unjust foils. Results are displayed in **Figures 2** and **3**.

As predicted in Hypothesis 2, in the unfair precue condition, there was a significant negative indirect effect of victim sensitivity on cooperative behavior in the trust game mediated by the endorsement of unjust targets, *B* = -3.68, *SE* = 2.28, 95% CI [-9.87; -0.53]. In the control condition, this indirect effect was not significant, *B* = 1.90, *SE* = 2.85, 95% CI [-1.52; 10.45]. Among persons who had been exposed to the unfair precue, higher (compared to lower) victim-sensitive persons endorsed unjust targets more strongly and, plausibly in consequence, reduced their behavioral cooperation.

Importantly, the endorsement of unjust targets was significantly related to reduced behavioral cooperation, *B* = -7.04, *SE* = 2.85, *p* = 0.02, which was not the case for the endorsement of unjust foils, *B* = -4.68, *SE* = 4.10, *p* = 0.26. Accordingly, there was no significant indirect effect of victim sensitivity on behavioral cooperation via the endorsement of unjust foils, neither in the unfair precue condition, *B* = -1.52, *SE* = 1.92, 95% CI [-8.07; 0.38], nor in the control condition, *B* = 0.49, *SE* = 1.12, 95% CI [-0.36; 5.11].

### Discussion

In Study 1 we adopted a moderated mediation approach to test whether victim sensitivity is related to the tendency to form expectancies of injustice, and whether this tendency mediates behavioral cooperation. Results suggested that victim sensitivity is related to a tendency to form expectancies of injustice under the unfair precue condition. The interaction of precue and victim sensitivity as well as the simple slope in the unfair precue condition were only significant regarding the endorsement of unjust targets, and not foils (Hypothesis 1A). Thus, our findings were compatible with the conclusion that, when the unfair precue had activated their suspicious mindset, highly (compared to lowly) victim-sensitive persons tended to form expectancies of unjust outcomes while reading the ambiguous scenario in the encoding phase. Our findings seem less compatible with the notion that JS involves a response bias manifested in the endorsement of unjust outcomes in the recognition phase, independent of whether these outcomes contradicted information in the initial scenario or not.

However, given a marginal relation between victim sensitivity and the endorsement of unjust foils in the unfair precue condition, it cannot be precluded that victim sensitivity is as well related to a response bias. For this reason, it was very important to test not only unjust targets, but also unjust foils as potential mediators on uncooperative behavior. These tests allowed us to decide whether expectancy tendencies (represented by the endorsement of unjust targets) and/or a response bias (represented by the endorsement of unjust foils) were responsible for the behavioral patterns of highly victim-sensitive persons.

In the unfair precue condition, the bivariate relation of victim sensitivity and behavioral cooperation in the trust game was not significant (also see, Rothmund et al., 2011). It is important to note that, despite the lack of a significant bivariate correlation, results were in accordance with our assumptions, as in the unfair precue condition there was a significant indirect effect of victim sensitivity on cooperation behavior via expectancies of unjust outcomes.

Specifically, this study provided the first evidence that the tendency in highly victim-sensitive persons to exhibit heightened expectancy of unjust outcomes contributes to the reluctance to cooperate in situations that require trust. Consistent with Hypothesis 2, in the unfair precue condition, there was a significant indirect effect of victim sensitivity on cooperation behavior mediated by the tendency to form expectancies of injustice. There was no indirect effect on behavior via the endorsement of unjust foils, indicating that a response bias was not relevant to the behavioral patterns of highly victim-sensitive persons.

## Study 2

The aim of Study 2 was to test the assumed causal relationship between the tendency to form expectancies of injustice and reduced cooperation behavior. We aimed to directly manipulate the mediator variable of Study 1 by means of a training procedure designed to induce differential expectancies concerning unjust vs. just outcomes, and we tested the effect of this manipulation on behavioral cooperation in the trust game.

### Method

#### Sample

Ninety-seven undergraduates (81% female; ages: 18–46 years; *M* = 21.30, *SD* = 3.49) participated in a study on language perception in return for partial course credit.

#### Procedure

Participants completed an online personality questionnaire that included the items to measure victim sensitivity (Schmitt et al., 2010) 2 weeks prior to their laboratory session. Upon arrival in the laboratory, participants were seated at one of four separated workplaces and randomly assigned to either the unjust expectancy training condition (*n* = 49) or the control condition (*n* = 48). They were instructed to work on a word fragment completion task that will be explained below. In the unjust expectancy training condition, this task aimed at inducing a readiness to form expectancies of injustice. Afterward, participants read ambiguous sentences and we assessed reaction times to word fragments that indicated the occurrence of unjust outcomes (unjust probe fragments), or just outcomes (just probe fragments), or outcomes unrelated to (in)justice (neutral probe fragments). In a subsequent trust game, we assessed cooperation behavior. Finally, participants were probed for suspicion and then debriefed, thanked, and dismissed.

#### Materials

##### Justice Sensitivity

We used the same scale as in Study 1 to measure victim JS (α = 0.89).

##### Expectancy training procedure

Twenty-four scenarios were constructed for use in the training condition that were ambiguous with regard to whether an unjust or just outcome would occur. Only the last words in some sentences determined which outcome occurred. These final words were presented as fragments that participants had to solve. In fact, every one of these fragments could be solved only to yield words that indicated the occurrence of an unjust outcome. Participants were instructed to use their understanding of the sentence and to press a marked button on the keyboard as soon as they knew the solution to complete the fragments. On the next screen, they typed in the missing letters and received feedback on their performance. After each fragment completion, a comprehension question followed. Participants answered “correct” or “false” by pressing the corresponding button as indicated by prompts on the screen. Half of the questions were accurately answered by the response “correct,” the other half by the response “false.” Again, participants received feedback after they responded to the question.

An example of a scenario from the unjust expectancy training condition is:

“In the cafeteria, three girls cut in line right in front of me. The cashier notices and serves the *g_rls f_ _st.*” (correct solution: girls first)

Twenty-four scenarios were constructed for use in the control version of this task. These materials were structured in a similar way, but unrelated to (in)justice.

An example of a scenario from the control version of the task is:

“In the cafeteria, a nice piece of cake catches my attention. The cashier notices and *recom_ _nds t_e cake.”* (correct solution: recommends the cake)

The assumption was that participants receiving the training would learn to readily form expectancies of an unfair outcome, through repeatedly completing fragments yielding unjust outcomes in the described scenarios. Thus, they should anticipate the unfair outcome of the sentence already during reading of the ambiguous passage. By contrast, in the control condition no such biased expectancy should be induced, given that the content of these scenarios was unrelated to (in)justice. The order of sentences and fragments was fixed across participants. Initially, three neutral practice trials were provided to assure the understanding of the instructions.

##### Assessment of expectancy tendencies

The induction of expectancy tendencies by the training procedure was measured with reaction times to solve additional fragmented sentences that followed the training procedure and the control condition in a fixed order. For the participants, no difference to the training material was apparent. They had to solve 12 probe fragments that ended ambiguous sentences, as with the previous fragments. However, different from the training task, four of these probe fragments indicated an unjust outcome, four a just outcome, and four further probe fragments resolved the ambiguous sentence in a way unrelated to (in)justice. These materials were used successfully by Baumert et al. (2012, Study 1, see also for examples) to assess biased expectancy of unjust vs. just outcomes. The scenarios preceding the unjust and just probe fragments were matched for length, and the fragments were matched in number of missing letters. We recorded how long participants took to press a marked button to continue to type in the missing letters. These reaction times were taken to reveal the degree to which expectancies of injustice (reactions times for unjust probe fragments) or justice (reaction times for just probe fragments) were formed when reading the ambiguous scenarios. The reaction times for neutral probe fragments served as baseline.

##### Trust game

We used the same trust game as in Study 1. As before, only the cooperation decision in the role of Person A was relevant for our research questions and will be reported in the results section.

To reiterate our hypotheses in Study 2, we predicted that participants in the unjust expectancy training condition would solve unjust probe fragments (but not just and neutral probe fragments) faster than participants in the control condition (Hypothesis 3). Furthermore, in the role of person A in the trust game, participants in the unjust expectancy training condition (compared to the control condition) should allocate less money to their partners (Hypothesis 4).

### Results

#### Justice Sensitivity

Participants did not differ significantly in their victim sensitivity between unjust expectancy training condition (*M* = 3.66, *SD* = 0.85) and control condition (*M* = 3.88, *SD* = 0.92), *t*(95) = -1.20, *p* = 0.23, *d* = 0.25.

#### Effects of Training on Readiness to Form Expectancies of Injustice

Before separately aggregating the reaction times for unjust, just, and neutral probe fragments, individual trials were omitted in which participants did not complete the fragment correctly. In general, error rates were very low for the different types of probes (3.35% for unjust; 2.83% for just; 2.35% for neutral probe fragments). In addition, we corrected for extreme outliers by omitting reaction times below 500 ms or above 15.000 ms (3.86% for unjust; 4.64% for just; 1.17% for neutral probe fragments).

To test the effectiveness of our training procedure we analyzed the reaction times for probe fragments using a 2 (training condition: unjust expectancy training/control) × 3 (probe type: unjust/just/neutral) ANOVA with repeated measures on the second factor. We found a significant main effect of probe type, *F*(2,94) = 17.99, *p* < 0.01, η^2^ = 0.28, and a significant probe type × training condition interaction effect, *F*(2,94) = 4.87, *p* = 0.01, η^2^ = 0.09. As expected (Hypothesis 3), participants in the unjust expectancy training condition came to solve unjust probe fragments significantly faster than did participants in the control condition (see **Table 2**), *t*(95) = -3.05, *p* < 0.01, one-tailed, *d* = 0.63. No such group difference was observed on reaction times to just probe fragments, *t*(95) = -0.59, *p* = 0.28, one-tailed, *d* = 0.12, or for neutral probe fragments, *t*(95) = -1.21, *p* = 0.12, one-tailed, *d* = 0.25. An *a posteriori* power analysis revealed 1-β = 0.78 for the interaction effect with η^2^ = 0.09, and 1-β = 0.92 for *post hoc t*-tests (one-tailed) with *d* = 0.63.

**Table 2:** Mean and standard deviation (in parentheses) of reaction times for unjust, just, and neutral probe fragments, and cooperation in the trust game, separately for the unjust expectancy training condition and control condition.

	Unjust expectancy training condition *M* (*SD*)	Control condition *M* (*SD*)
Unjust probe fragments	3636 (999)	4352 (1297)
Just probe fragments	4094 (1157)	4240 (1243)
Neutral probe fragments	4447 (1011)	4713 (1150)
Cooperation trust game	65.4 (30.8)	75.3 (23.5)

*Unjust probe fragments = mean reaction times for unjust probe fragments (ms). Just probe fragments = mean reaction times for just probe fragments (ms). Neutral probe fragments = mean reaction times for neutral probe fragments (ms). Cooperation trust game = investment in the role of Person A (in Cent) in the trust game*.

#### Trust Game

Participants in the role of Person A on average transferred 70.4 ct (*SD* = 27.6) of their initial amount of 100 ct to their anonymous partner. Again, the value of this monetary transfer was our measure of behavioral cooperation. As predicted (Hypothesis 4), there was a significant difference between the conditions, *t*(95) = -1.78, *p* = 0.04, one-tailed, *d* = 0.37, with participants in the unjust expectancy training condition transferring less money to their interaction partner than did participants in the control condition (**Table 2**). For this effect with *d* = 0.37 the power analysis revealed *1-*β = 0.57.

### Discussion

The goal of Study 2 was to experimentally induce a group difference in the readiness to form expectancies of unjust outcomes in ambiguous situations by means of a training procedure, in order to determine the causal impact of this expectancy tendency on cooperation behavior.

Participants who received the unjust expectancy training became faster (compared to the control condition) to complete word fragments that resolved the ambiguity by indicating an unjust outcome. There were no differences between the conditions in the reaction times to word fragments indicating just outcomes, or outcomes unrelated to (in)justice (Hypothesis 3). This represents evidence that participants in the training condition learned to readily form expectancies of unjust outcomes in ambiguous situations. Importantly, we found that participants in the unjust expectancy training (compared to the control condition) were significantly less cooperative in the trust game in the role of Person A (Hypothesis 4). Having learned to readily expect unjust outcomes in ambiguous situations, participants likely expected their anonymous interaction partner to exploit them and, consequently, reduced their cooperation. Importantly, this study provides a rigorous test of the assumption that the readiness to form expectancies of unjust outcomes can causally contribute to reduced behavioral cooperation in circumstances that require trust in others.

## General Discussion

We investigated how dispositional victim sensitivity shapes cooperation behavior, to test the validity of hypotheses derived from the SeMI model (Gollwitzer et al., 2013). We tested the notion that victim-sensitive persons have a disproportionate tendency to form expectancies that others harbor mean intentions, and that this pattern of expectancies causally contributes to their withdrawal of cooperation in socially uncertain situations.

In Study 1 we found evidence in support of central assumptions of the SeMI model, namely that under conditions that cue activation of a suspicious mindset, information processing of high victim-sensitive persons is characterized by the tendency to form expectancies of injustice. In line with Hypothesis 1A, we found highly (compared to lowly) victim-sensitive persons who had previously experienced an unfairness exhibited stronger expectancy for unjust outcomes. When activation of a suspicious mindset was not cued by prior unfairness, there was no effect of victim sensitivity on expectancy for unjust outcomes.

In sum, these findings suggest that people high in victim sensitivity exhibit a heightened tendency to form expectancies of injustice in specific situations. However, it cannot be completely ruled out that highly victim-sensitive persons might be (additionally) characterized by a tendency to regard the world as a generally unjust place. In other words, they might show a response bias, since there was a marginally significant relationship to the endorsement of unjust foils in the unfair precue condition. Yet, findings of the moderated mediation analyses showed that only expectancies of injustice of highly victim-sensitive persons causally contributed to their reduced cooperation. Consistent with Hypothesis 2, there was a significant indirect effect of victim sensitivity on cooperation behavior in the unfair precue condition, and this association was mediated by the tendency to form expectancies of injustice. Apparently, in the trust game, victim-sensitive persons tended to expect an unjust outcome and, in turn, avoided to make themselves vulnerable for exploitation.

This result might contribute important knowledge to the current debate about the acceptance and solidarity for refugees coming to Europe. It can be expected that Europeans high in victim sensitivity will be particularly reluctant to show solidarity and to welcome refugees, because they are suspicious that their benevolence could be exploited and that they would suffer therefore deprivation later on. Future studies are necessary to investigate this important psychological mechanism in similar phenomena that occur in daily life.

Interestingly, in the control condition there was a significant positive correlation between victim sensitivity and behavioral cooperation (see **Table 1**), but there was no evidence that this was mediated by expectancy tendencies. One might speculate that fairness concerns are decisive in this situation. Possibly, the prior experience of a fair outcome may have suppressed the activation of the suspicious mindset. Gollwitzer et al. (2005) argued that highly victim-sensitive persons might experience a motivational conflict when their suspicious mindset is activated: they have a strong concern for justice, but since exploitation is particularly aversive for them, they have also a strong fear of being exploited. Future studies could investigate this issue by testing if the suppression of the suspicious mindset mediates the positive association between victim sensitivity and prosocial behavior in situations where fair treatment has been experienced.

To enrich these findings and to identify causal relationships, we induced expectancies of unjust outcomes in ambiguous situations, in order to test the causal impact of such expectancies on behavioral cooperation. This is an important contribution to understand the underlying processes of cooperation behavior, since correlation evidence from Study 1 shows only that expectancy tendencies are associated with this kind of behavioral reactions. Without the experimental manipulation of expectancy, it would not be possible to distinguish alternative causal accounts of the observed mediation effect, such as the reversed pattern of causality with frequently displayed uncooperative behavior shaping expectancies regarding the likelihood of unfair outcomes, which in turn might influence levels of victim sensitivity over time.

Specifically, results suggest that reduced cooperation in the trust game, observed among victim-sensitive persons (e.g., Gollwitzer and Rothmund, 2011) can be induced by manipulating expectancy tendencies. We provided strong evidence for the assumed causal relationship. The observed impact of our training procedure indicated that there is potential to modify tendencies to form expectancies of injustice in ambiguous situations by means of specific interventions that could have therapeutic application. Thus, we have laid the foundation for training-based interventions to enhance cooperation by directly targeting for change in the underlying cognitive processes that translate this disposition into potentially maladaptive behavior.

It remains to be seen whether directly training increased expectancy of just outcomes would result in the enhancement of cooperation in real world settings. Testing the causal impact of a just expectancy training condition on cooperation behavior will be a meaningful first step. Further research should determine if the tendency to expect injustice displayed by high victim-sensitive persons can be permanently reduced through extended training to yield a beneficial increase in cooperative behavior. One study that has taken a preliminary step in this direction showed that participants acted more prosocially in an economic game after being trained to expect own unjustified advantages (Maltese et al., 2013). Highly victim-sensitive persons may benefit from a similar prosocial training that directs their thoughts on disadvantaged others and away from the expectancy of own disadvantages resulting from the perception of others mean intentions.

On a more general level, our results emphasize the importance of illuminating the patterns of information processing that translate latent dispositions into overt behavior. Until now, most studies tested only bivariate correlations between personality traits and information processing patterns (e.g., Rusting, 1998). Importantly, we substantially extended this approach by investigating moderated mediations, showing how the interaction between personality and situation shapes information processing which leads to differences in behavioral reactions. Furthermore, we were also able to show the causal relationship between the information processing of victim-sensitive persons and their cooperation behavior. This approach should encourage further studies in social justice research, as well as in other areas of psychology.

### Limitations

Despite their strengths, our studies also have certain limitations. First, we tested behavioral cooperation only in the trust game. Thus, it remains to be seen whether our results generalize to cooperative behavior in social dilemma situations (e.g., Gollwitzer et al., 2009), in public goods games (e.g., Hilbig et al., 2012), or in real-life situations where cooperation is required (e.g., solidarity with refugees). In all these situations, the identified pattern of activation of a suspicious mindset and formation of expectancies concerning the prospect of just vs. unjust outcomes should be relevant as mediator for behavioral reactions.

Second, the methods employed to assess and manipulate expectancies of injustice in Studies 1 and 2 were not identical. In Study 1, we measured the tendency to form expectancies of unjust outcomes by assessing the endorsement of targets indicating unjust outcomes for previously encoded scenarios. To experimentally induce a tendency to expect unjust outcomes in Study 2, we used a fragment completion training task, and assessed the impact of this training on expectancy by measuring reaction times to solve fragmented words indicating just and unjust outcomes for events described in preceding sentences. Such differences between the tasks introduce the possibility that in Study 2 we may have manipulated somewhat different expectancy processes from those we measured in Study 1. Nevertheless, we believe our results speak in favor of the option that assessment and manipulation tapped into similar tendencies, showing the importance of expectancies of injustice for reactions in the trust game.

Third, while we have shown that the heightened tendency of highly victim-sensitive persons to form expectancies of injustice mediates the association between victim sensitivity and reduced behavioral cooperation, it is plausible that other processes also may contribute to the mediation of this relationship. It would be fruitful for further studies to investigate possible other mediating processes (e.g., availability of legitimizing cognitions) to extend understanding of the pathways through which victim sensitivity serves to compromise cooperative behavior.

Fourth, the power for the effects of victim sensitivity on expectancies of injustice and of the expectancy training on cooperation behavior did not correspond to the conventions of *1-*β = 0.80. As has been shown, underpowered studies may lead to an increased likelihood of false positive findings (Button et al., 2013). For this reason, a replication with a bigger sample size is necessary in order to raise confidence in the robustness of the revealed effect.

## Conclusion

In two studies we provided evidence for hypotheses derived from the SeMI model that describes the underlying processes translating dispositional victim sensitivity into reduced cooperation behavior. Our findings suggest that in highly victim-sensitive persons the activation of a suspicious mindset in response to perceived unfairness increases the tendency to form expectancies of injustice, which mediates the withdrawal of behavioral cooperation. By manipulating the tendency to form expectancies of injustice we were able to show that such expectancy exerts a causal influence on cooperation behavior. These findings enrich the theoretical conceptualization of the SeMI model, and extend application for social justice research in ways that could be fruitful for other areas of psychology. These studies also pave the way for future applied research designed to enhance cooperative behavior in people with high levels of victim sensitivity.

## Conflict of Interest Statement

The authors declare that the research was conducted in the absence of any commercial or financial relationships that could be construed as a potential conflict of interest.

## Funding

This research was supported by a grant from the German Research Foundation, No. SCHM1092-10/3.

## Footnotes

1 Several other personality measures were administered. These are not relevant for the present research questions and, thus, will not be analyzed in the present context.

2 Originally, these sentences were designed to assess spontaneous interpretations of ambiguity by means of reactions times. Participants had to complete fragments that were intended to yield either an unjust, just or neutral outcome (10 each) of an ambiguous sentence. However, in an independent sample, fairness and valence ratings of the supposedly unjust, just, and neutral outcomes were not sufficiently distinct. Therefore, we excluded this task from further analyses. Materials are available upon request.

3 It was counterbalanced across participants which specific scenario was structured in a way that allowed to assess expectancy tendencies. For half of the participants, six scenarios (for the other half 7) were ambiguous with regard to the outcome for the narrator. The other scenarios reported negative outcomes for the narrator thus not serving for the assessment of expectancy tendencies.

4 The 52 outcome sentences for all 13 relevant scenarios were pretested by nine independent raters (ages: 21–78 years; *M* = 37.1; *SD* = 21.3). Response scales ranged from 1 (*very unjust*) to 6 (*just*). Unjust outcomes were rated significantly lower (*M* = 1.38; *SD* = 0.55) than just outcomes (*M* = 4.72; *SD* = 0.72), *t*(8) = -8.52, *p* < 0.01*, d* = 5.67. When comparing targets and foils, in two scenarios ratings differed more than 2 *SD* from each other. Therefore, all sentences belonging to these scenarios were excluded from further analyses. For the sentences belonging to the remaining 11 scenarios, unjust targets (*M* = 1.33; *SD* = 0.64) were rated similar to unjust foils (*M* = 1.43, *SD* = 0.52), *t*(8) = -0.90, *p* = 0.40, *d* = 0.18, and just targets (*M* = 4.70; *SD* = 0.91) were rated similar to just foils (*M* = 4.73; *SD* = 0.61), *t*(8) = -0.17, *p* = 0.87, *d* = 0.04. The main results of the study remained similar, when including or excluding the two scenarios.
